# Comparative Longevity of Bryophyte Spores: Influence of Storage Conditions, Spore Maturity, and Plant Ecology

**DOI:** 10.3390/plants15152309

**Published:** 2026-07-27

**Authors:** Belén Albertos, Aleksandra Ruzic, Ricardo Garilleti, Francisco Lara, Daniel Ballesteros

**Affiliations:** 1 Departamento de Botánica y Geología, Universitat de València, 46100 Burjassot, Spain; belen.albertos@uv.es (B.A.); ricardo.garilleti@uv.es (R.G.); daniel.ballesteros@uv.es (D.B.); 2 Departamento de Biología, Facultad de Ciencias, Universidad Autónoma de Madrid, 28049 Madrid, Spain; francisco.lara@uam.es; 3 Centro de Investigación en Biodiversidad y Cambio Global, Universidad Autónoma de Madrid, 28049 Madrid, Spain; 4 Jardí Botànic de la Universitat de València, 46008 Valencia, Spain; 5 Royal Botanic Gardens Kew, Wakehurst, Ardingly RH17 6TN, West Sussex, UK

**Keywords:** accelerated ageing, dry storage, spore longevity, P_50_, survival curves

## Abstract

Bryophyte spores are known for long-distance dispersal and tolerance to adverse conditions, but their comparative longevity remains poorly understood. In this work we assessed whether moss spores are as long-lived as spores and seeds of other land plants. Spores of *Funaria hygrometrica*, *Lewinskya acuminata*, *L. iberica* and *Ulota crispula* were subjected to accelerated aging (AA: 45 °C, 60% relative humidity [RH]) and dry storage (15% RH, 20 °C) with periodic germination tests. Different maturity stages of *F. hygrometrica* were also included. Estimated initial viability constant (Ki), standard deviation of spore deaths’ distribution in time (σ) and time for viability to drop to 50% (P_50_) were determined by probit analysis from spore survival curves. Overall results showed short spore longevity for all species tested, regardless of storage conditions. P_50_ ranged from 0.4 to 5.7 days (AA) and 58–150 days (dry storage), indicating faster deterioration than seeds or fern spores at the same storage conditions. In the case of *F. hygrometrica*, spores collected at peak maturity showed the longest longevity under both storage conditions. This study suggest that moss spores may be relatively short-lived, which could be related to their chlorophyllous nature. Ecological and conservation implications of this spore character are discussed.

## 1. Introduction

As the degradation of natural ecosystems and habitats continues to pose an increasing threat to our planet’s rich biodiversity, there is a growing need to implement ex situ conservation measures in conjunction with our in situ efforts [1]. This strategy serves as both a temporary solution and a contingency, ensuring the conservation of genetic material in worst-case scenarios. Within the ex situ conservation approaches, seed banking is a widely adopted method [1]. This practice entails storing dry seeds at low temperatures, a set of controlled conditions that prolong their viability over a large period of time. At such conditions, seeds of some species can exhibit remarkable longevity, with the ability to remain viable for hundreds or even thousands of years [2,3,4].

Seed longevity is a complex trait that varies among and within species and depends on the interaction of the physiological and physical–chemical characteristics of the seed with the surrounding environment [5,6,7]. Understanding seed longevity contributes to enhancing the efficiency of species management within a seed bank. This knowledge enables us to make more accurate predictions, including the duration of intervals before stored seeds necessitate re-testing for viability or determining the timing for replenishing seed stocks to maintain a high level of viability [6].

One established method for assessing the longevity of seeds involves determining their half-life, i.e., the duration required for viability to decrease to 50% (referred to as P_50_) [4,8]. However, directly measuring seed longevity by observing real-time viability decay can become impractical. For example, seeds stored at the low humidity and temperatures of a seed bank may last for decades or centuries [3,9]. To overcome this issue, a standardized procedure has been developed involving controlled exposure of the seeds to high humidity and temperature (usually a relative humidity of 60% at 45 °C, e.g., [10]). This process facilitates the accelerated degradation of seed viability, thereby enabling the rapid determination of P_50_ values for comparative purposes in the laboratory [11,12,13].

While significant research efforts have been dedicated to deciphering seed longevity and improving their ex situ conservation [1,3,6,7,14,15], the longevity of other plant propagules, such as bryophytes spores, has been overlooked in this aspect. Knowledge about spore longevity is fragmentary even for thoroughly studied species such as the model organisms *Funaria hygrometrica* Hedw. and *Physcomitrium patens* (Hedw.) Mitt. [16]. This lack of knowledge represents a gap not only for improving their ex situ conservation, but also for understanding ecological features like plant dispersal and propagule persistence [17,18,19]. In terms of conservation, the latest IUCN report identified bryophytes [20] as the third most threatened group of plant species, with documented regional extinctions in Europe. The primary threat to bryophytes stems from anthropogenic activities, particularly habitat destruction and territorial alterations resulting from agricultural practices, dam construction, and related activities. These threats are further aggravated by the consequences of climate change. Consequently, there has been a surge in research on bryophytes in recent years, along with initiatives to integrate them into conservation efforts [21,22].

Bryophytes differ significantly in structure and evolution from other vascular plants. They are characterized by a haplo-diplophasic life cycle with a dominant gametophytic phase [23] and disperse via spores instead of seeds. Moreover, seeds (diploid and multicellular) and spores (haploid and unicellular) represent different structures within the life cycle of the organisms [24,25]. However, both seeds and spores share diverse physiological attributes that makes them analogous in terms of longevity. Indeed, spores of other land plants, such as ferns, have been extensively used as unicellular systems to approach longevity issues observed in seeds [26,27]. Therefore, bryophyte spore longevity could be better understood if studied using the extensive knowledge and approaches developed for the study of longevity for seeds (and spores) of vascular plants.

Bryophyte spores exhibit great resistance to adverse conditions [18] and because of their small size, they can be carried over long distances by wind currents [28,29], contributing to their ecological success in diverse terrestrial environments (ranging from tropical to polar regions and the sub-Antarctic) [30]. However, there still exists a lack of empirical evidence regarding the longevity of their spores [19,28,29,31,32,33]. Despite the limited research, various observations suggest the enduring viability of bryophyte spores over extended periods. Bryophytes are thought to produce long-lasting spores, as evidenced by cases where spores, stored for significant durations, retained their viability. In germination tests conducted by [28], spores were found to remain viable for 1–3 years when desiccated and kept in frozen conditions. Earlier studies by Malta [32,33] demonstrated the germination of *Ceratodon purpureus* (Hedw.) Brid. after 16 years in a herbarium and *Funaria hygrometrica* after 13 years. Meyer [34] also found *F. hygrometrica* to be viable after 8 years of storage in xeric conditions. More recent findings include the discovery of viable *Sphagnum* spores centuries old in peat cores (waterlogged, anoxygenic environment) [35] and the finding of viable diaspores of *Physcomitrium eurystomum* Sendtn. in sediment layers several hundred years old [36].

While much research has focused on peatlands [35,37,38] and cryogenic environments [39], where spores can survive for centuries due to exceptional environmental conditions, less is known about their persistence in mineral or non-peat wet soils at temperatures above zero (but see [40,41,42,43,44]). Even in peatland spore banks, the viability of *Sphagnum* spores is proven to be limited, with a half-life of between one and 26 years, and generally less than 10 years [45,46]. However, there is significant variation among cohorts and capsules, possibly due to environmental and maternal effects [45,47], which could explain the instances of exceptional longevity.

While these observations suggest that long-term storage might be a viable option, they also indicate a high dependence on specific preservation conditions and do not provide quantitative information on how frequently this extreme longevity occurs among the spores generated by a population. Therefore, more comparative evidence is needed to confirm the presumed extended lifespan of bryophyte spores across species and storage conditions. Planned experimentation, incorporating quantitative measurements, is necessary in a comparative manner to determine the potential longevity of spores.

Hence, the primary goal of this paper was to provide a comparative methodological approach to test the hypothesis that bryophyte spores have a relative long lifespan. For the completion of this research, we employed an accelerated aging procedure developed for seeds [10] on bryophyte spores for the first time. Then, the acquired data was compared with that available for other spores (ferns) and seeds (angiosperms) in the same aging context. Additionally, we sought to investigate whether the longevity of bryophyte spores is influenced by their maturity stage. We hypothesize that, akin to the pattern observed in seeds of vascular plants, longevity will increase as the spore maturity increases.

For this purpose, we made an initial selection of four bryophyte species: a cosmopolite and pioneer species with spores reported to survive long periods of time in herbarium conditions, *Funaria hygrometrica* [33,34], and three epiphyte species of the Orthotrichaceae family with a presumably short spore lifespan (less than 6 years [33] and personal observation). Three experiments were conducted with these species: (1) longevity of mature spores of *Ulota crispula* Bruch., *Lewinskya iberica* (F.Lara & Mazimpaka) F.Lara, Garilleti & Goffinet and *L. acuminata* (H.Philib.) F.Lara, Garilleti & Goffinet, stored under accelerated aging conditions; (2) longevity of *F. hygrometrica* spores at different degrees of maturity, stored under accelerated aging conditions; and (3) longevity of *F. hygrometrica* spores at different degrees of maturity stored under dry conditions. All three experiments share a similar procedural approach, differing only in the storage conditions and the specific spores studied. We expect that this comparative methodological approach will constitute an initial database on longevity parameters of bryophyte spores that will be increased in future works across bryophyte lineages.

## 2. Results

### 2.1. Longevity of Bryophyte Spores

**Figure 1:**
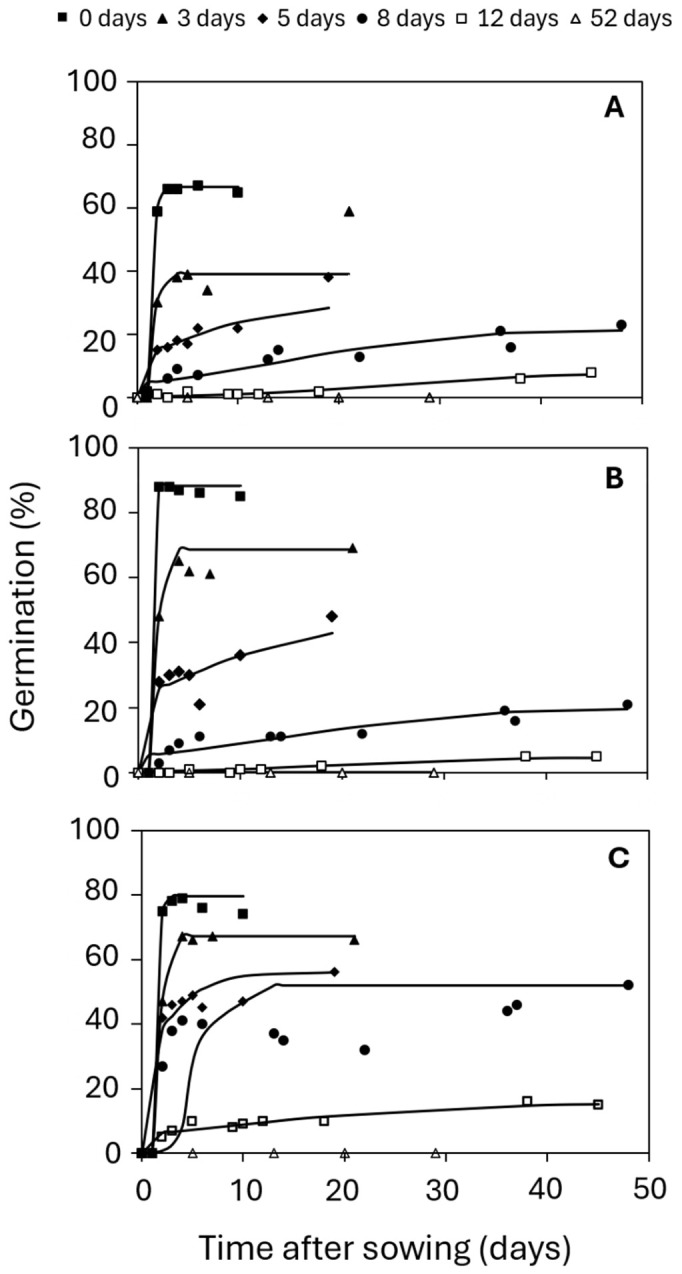
Germination curves for experimentally aged *Funaria hygrometrica* spores at different maturity stages: (**A**) most immature spores in green capsules, (**B**) near-full maturity spores in yellow capsules, (**C**) fully mature spores in brown capsules. In each curve, symbols represent the proportion of germination over time measured for each aging time assayed (indicated with different symbols). The resultant germination curves (solid lines) follow a sigmoidal distribution obtained after fitting the experimental points of each aging time to a Boltzmann curve.

**Figure 2:**
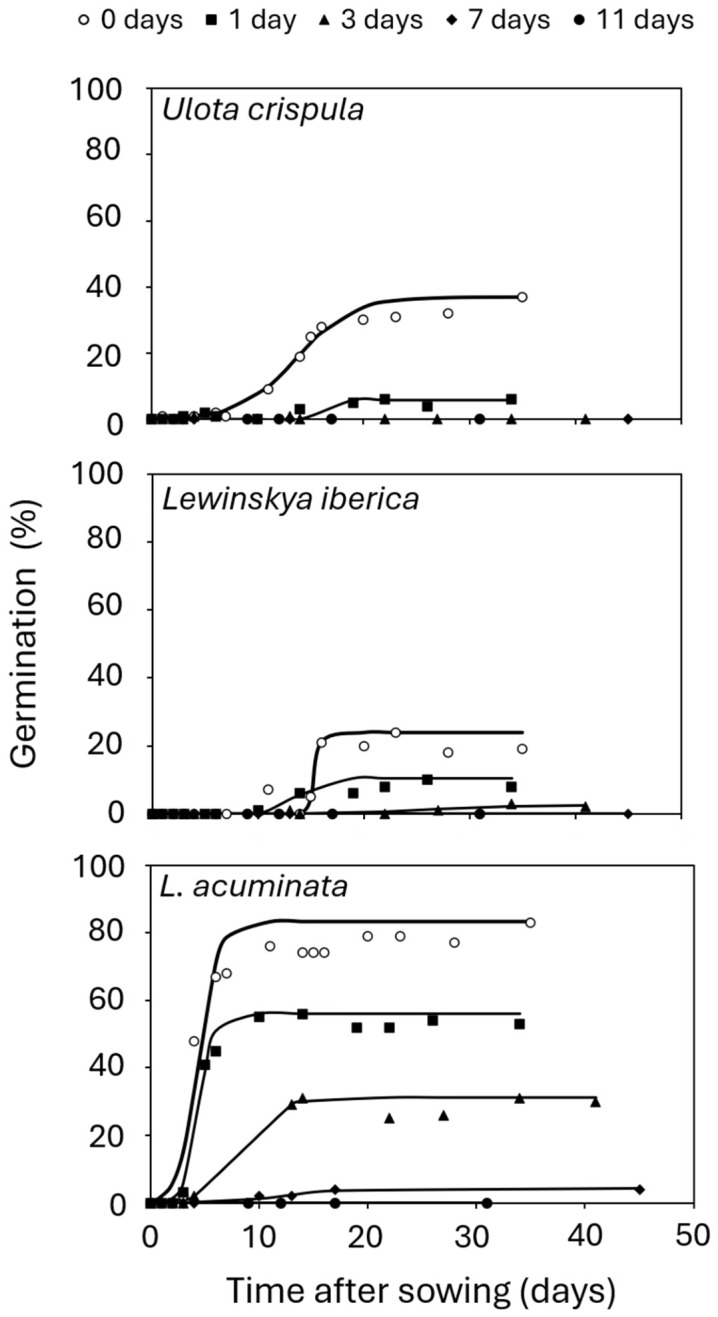
Germination curves for *Ulota crispula*, *Lewinskya iberica* and *L. acuminata* spores that were experimentally aged. In each curve, symbols represent the proportion of germination over time measured for each aging time assayed (indicated with different symbols). The resultant germination curves (solid lines) follow a sigmoidal distribution obtained after fitting the experimental points of each aging time to a Boltzmann curve.

All spores that were experimentally aged experienced a gradual decrease in their viability as the time they spent in storage was prolonged. The percentage of germinated spores counted each day after sowing was plotted for each species, resulting in different sigmoidal curves for each time of storage in accelerated aging conditions that were fitted with the Boltzmann distribution (Figure 1 and Figure 2). As storage time increased, the final percentage of germinated spores gradually declined. Additionally, equilibrium—representing the final germination percentage—was reached more slowly with prolonged storage duration.

**Figure 3:**
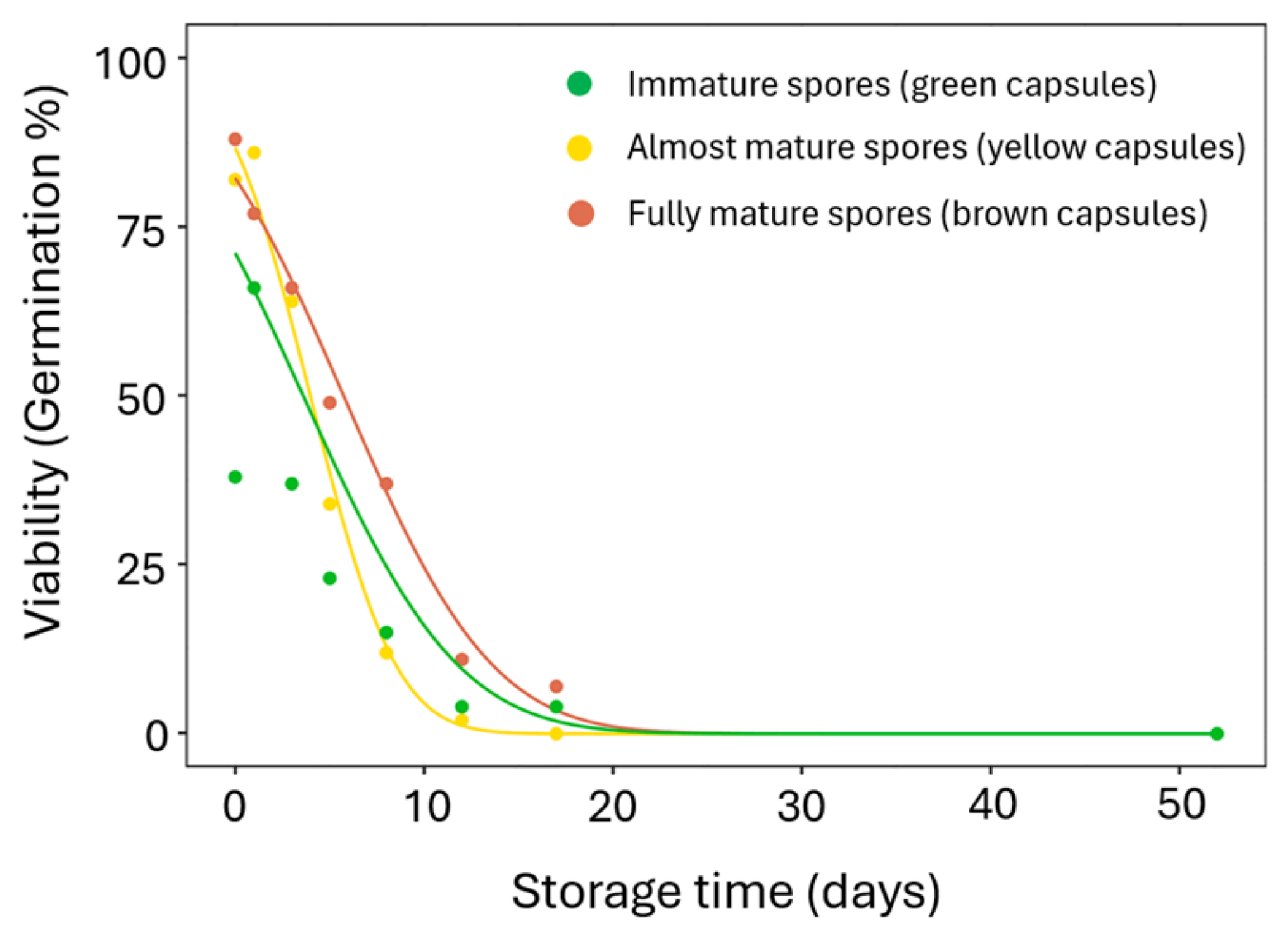
Survival curves fitted to a GLM with a probit link for the three maturity stages of *Funaria hygrometrica* spores stored at 60% RH and 45 °C. Green dots and lines represent the most immature spores collected from green capsules, yellow dots and lines are for the almost mature spores collected from yellow capsules, and brown dots and lines are for the fully mature spores collected from brown capsules.

**Figure 4:**
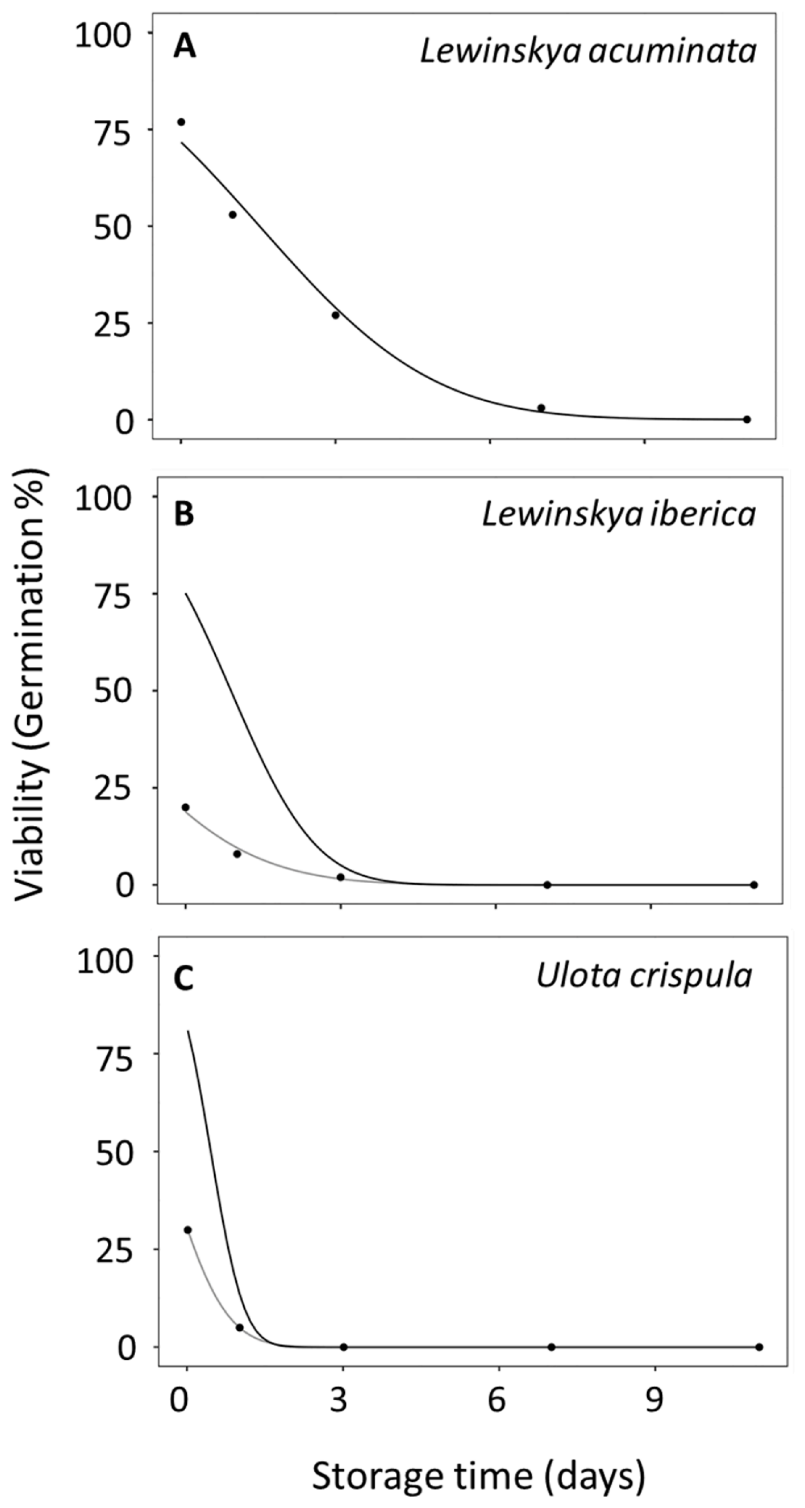
Survival curves for *Lewinskya acuminata* (**A**), *L. iberica* (**B**), and *Ulota crispula* (**C**). All curves were fitted to a GLM with a probit link. The black curve represents the fitted equation where a very low (<40%) initial viability was taken into account. The grey curve represents the normally fitted curve.

Full loss of viability (0% germination) was achieved relatively fast in all species, between 1 and 4 weeks, but at different rates depending on the species (Figure 3 and Figure 4). For example, *Funaria hygrometrica* needed ca. 20 days in accelerated aging to show full viability loss (Figure 3). On the other hand, *Lewinskya acuminata* lost all viability between 7 and 10 days of accelerated aging (Figure 4A), and *L. iberica* and *Ulota crispula* lost all viability before 4 days of accelerated aging (Figure 4B,C).

The different aging rates are also indicated by the σ and the P_50_ values of the Ellis and Robert viability equations, as summarized in Table 1. For example, P_50_ values recorded for our species ranged from 0.4 days for *Ulota crispula* to 5.7 days for the brown spores of *Funaria hygrometrica*; the latter species showed a greater longevity for the three maturations tested compared to the other three species (GLM, ANOVA, *p* < 0.05). A comparison between the P_50_ values of the species revealed that *F. hygrometrica* was significantly different (ANOVA + Tukey’s HSD, *p* < 0.05) to the other species tested.

### 2.2. Effect of Dry Storage Conditions on Spore Longevity

**Figure 5:**
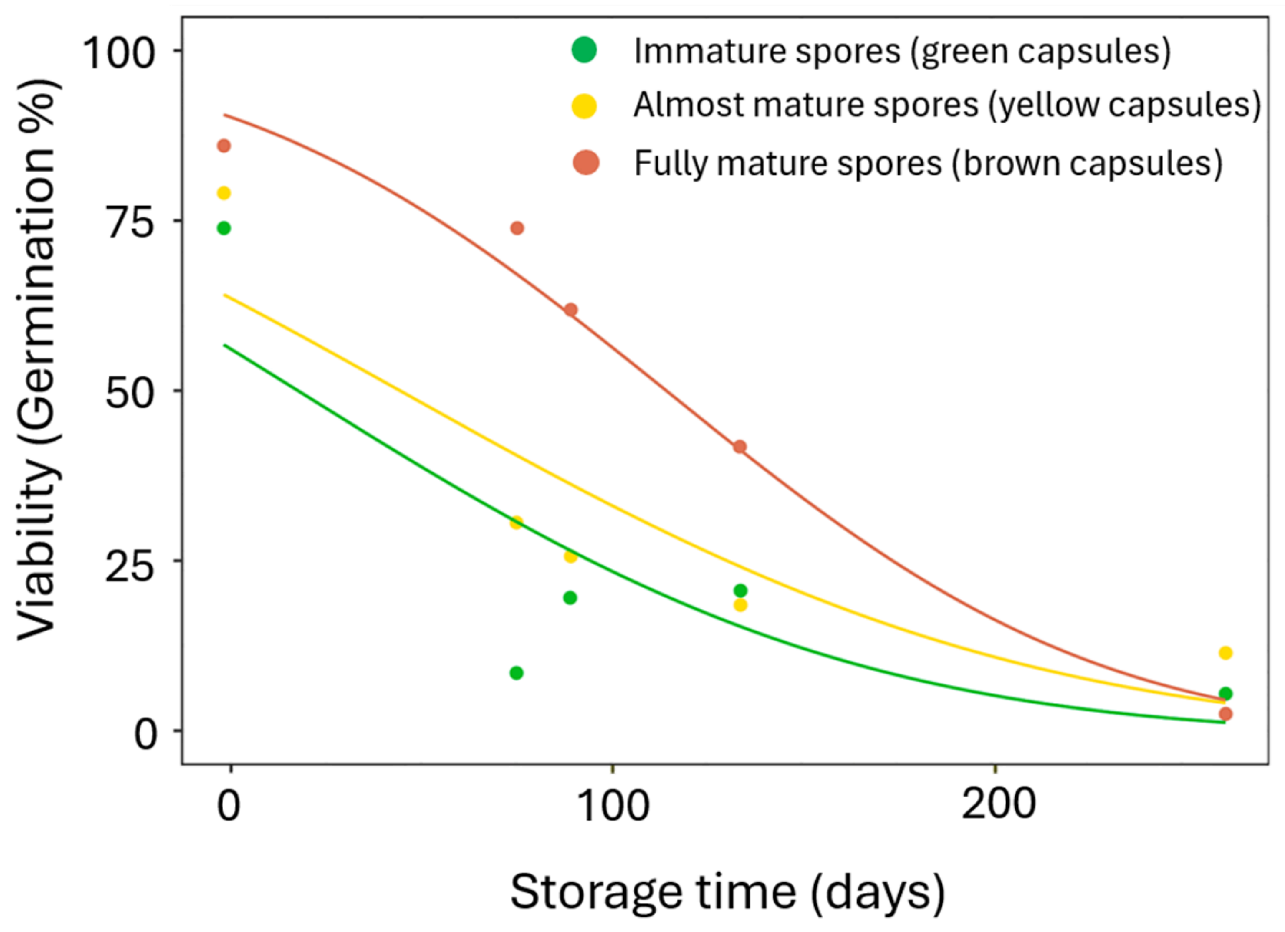
Survival curves fitted to a GLM with a probit link for the three maturity stages of *Funaria hygrometrica* stored at 15% RH and 20 °C (dry conditions). Green dots and lines represent the most immature spores collected from green capsules, yellow dots and lines are for the almost mature spores collected from yellow capsules, and brown dots and lines are for the fully mature spores collected from brown capsules.

All the *Funaria hygrometrica* spores that were stored in dry conditions experienced a gradual decrease in their viability, without ever reaching 0% in the time measured; nonetheless, we were able to calculate the P_50_ values for each maturity stage. The results showed that the spores remained viable far longer compared to the controlled aging experiment, with green (immature) spores showing the least longevity at 58.2 days and brown (mature) spores with the highest longevity at 150.6 days (Figure 5, Table 2).

With an ANOVA followed by Tukey’s HSD we found that there was a significant difference (*p* < 0.05) between the brown spores compared to the other two maturity stages. On the other hand, when we applied the same test to the *F. hygrometrica* spores that underwent accelerated aging we found that the maturity stage did not show any significant effect on spore viability (ANOVA, *p*-value = 0.321).

### 2.3. Comparison of Bryophytes with Other Plant Systems

We compared the P_50_ values of our bryophyte spores with those of seeds or spores of vascular plants (flowering plants and ferns) subjected to the same accelerated aging procedure (Table 3). Our calculated P_50_ values for bryophyte spores fell within the lower range of seed longevities, with *Streptocarpus cyaneus* at the lower end (P_50_ of 0.9 days) and *Caiophora latenitia* at the upper end (P_50_ of 4.3 days). *Funaria hygrometrica* exhibited a slightly higher P_50_ value at 5.70 days.

Concerning bryophyte spores stored in dry conditions, their P_50_ values were compared to those of fern spores (Table 3). The P_50_ values of fully mature *F. hygrometrica* spores (i.e., brown capsules, P_50_ = 150.6 days) were far below those calculated for non-chlorophyllous fern species and were closely aligned with that of the relatively short-lived chlorophyllous spores of the fern *Onoclea struthiopteris* (≡ *Matteuccia struthiopteris*), registered at 119 days. Spores from green and yellow capsules presented lower P_50_ values (58 and 80, respectively) than *O. struthiopteris*, but higher values than the very short-lived spores of *Todea barbara*.

## 3. Discussion

Our objective was to investigate the longevity of bryophyte spores from a diversity of species, also examining potential variations based on the maturity stage. Contrary to the hypothesis, the P_50_ values for our four species indicated transient or very short-lived longevity. This observation was reinforced by comparing our results with other plant systems that underwent experimental aging following the same procedure. The longevity of bryophyte spores can be extended through storage in dry conditions, with the maturity stage of the spore emerging as a significant factor influencing its longevity. Here we discuss the implications of longevity for ex situ bryophyte conservation and the persistence of in situ soil spore banks.

### 3.1. Comparative Longevity of Bryophyte Spores

The spores of the species we examined underwent storage conditions of 60% RH at 45 °C, with the aim of accelerating the aging process to derive the P_50_ values for comparative purposes. Our findings suggest that compared to other plant systems, bryophyte spores may exhibit very short life spans. For example, when we compare the P_50_ values of our bryophyte spores with those of seeds of vascular plants subjected to the same accelerated aging conditions (Table 3, [11]), we could reasonably conclude that our bryophyte spores exhibit notably brief lifespans. For instance, the mean P_50_ values of both endospermic and non-endospermic seeds was 18.17 and 63.57 days, respectively [11]. In all our bryophyte species their spores exhibited P_50_ values below 6 days (Table 1), which are far below the mean P_50_ values indicated above. Notably, the three epiphytic species displayed P_50_ values between 0.4 and 1.5 days, while *F. hygrometrica* spores at all three maturation levels showed P_50_ values between 3.61 and 5.70 days (Table 1), which roughly correspond to those P_50_ values observed for very short-lived seeds from the species like *Streptocarpus cyaneus* and *Caiophora lateritia*, respectively (Table 3). The results in spores under accelerated aging conditions are also supported by the results of the *F. hygrometrica* spores at all three maturation levels aged under dry storage conditions. Specifically, P_50_ values of *F. hygrometrica* spores aged at 15% RH and 20 °C ranged between 58 and 150 days, which falls within the range of the P_50_ values for chlorophyllous fern spores such as *Todea barbara* and *Onoclea struthiopteris*; both are considered short-lived when compared with non-chlorophyllous fern spores that range between approx. 300 and 4800 days (Table 3, [26,50]). However, our results contradict those observed in spores of *F. hygrometrica* stored dry (23 °C, 25–45% RH) in a herbarium, which retained germination rates between 33 and 22% after 4.6 and 28 years of storage, respectively [51]. This could be related to longevity differences between *F. hygrometrica* populations stored in the different experiments, indicating large intraspecific variation in spore longevity. Another explanation could be related to different storage conditions affecting longevity. For example, spores in [51] were stored in continuous dark conditions, while our spores were stored in a room with light for at least 10–12 h per day. In this sense, light exposure could have increased the aging rate of our *F. hygrometrica* spores through oxidative stress, as it has been described in previous research for dry seeds and fern spore during dry storage [7]. In conclusion, our findings suggest that bryophyte spores are in many cases short-lived. Nonetheless, more comparative long-term studies with diverse species and populations of the same species in the same storage conditions are needed, while also adding physiological measures to determine the causes of the longevity trait.

In terms of soil persistence, traditionally, seed persistence in the soil is categorized into three groups: transient (<1 year), short-lived (1 to 5 years) and long-lived (>5 years). The authors of [8,52] conducted a study in which they validated the use of accelerated aging techniques by finding a positive correlation between P_50_ values and the persistence of vascular plant seeds in soil. They categorized P_50_ values into three interpretations: <20 days for seeds persisting <1 year in soil (transient or particularly short-lived), 20 to 50 days for seeds persisting 1 to 3 years in soil (considered short-lived), and >50 days for seeds persisting >3 years in soil (long-lived). A more conservative approach to classifying short-lived seeds [12] considered P_50_ values <2 days as transient/ephemeral and those of 2 to 10 days as very short-lived. If we apply these categorizations to bryophyte spores and considering that our four species exhibited P_50_ values below 6 days, the spores of all of them would be considered very short-lived, falling within the transient persistence category.

Our results indicate a low spore longevity for the studied species, suggesting a limited capacity to form long-term spore banks. This pattern is consistent with previous studies showing that bryophyte spore banks do not necessarily preserve the full composition of above-ground assemblages [53], implying that many species rely more on dispersal than on persistent soil diaspore banks, as proposed for small-seeded plants [54] and for fugitive and colonist bryophyte species such as *Funaria hygrometrica* [41].

In the case of *F. hygrometrica*, although 13 to 28 years of viability under herbarium conditions [33,51] have been reported, field studies do not support the formation of persistent soil spore banks. This species was absent from spore banks in chalk grasslands in the Netherlands [40,41] and was not detected in sediment cores from a fish pond where it co-occurred with other Funariaceae [16]. In contrast, viable spores of *Physcomitrium patens* and *P. eurystomum* were recovered, the latter being among the longest-lived bryophyte spores recorded [36]. These findings reinforce the idea that not all bryophytes possess sufficient spore longevity to build persistent soil banks, in agreement with our accelerated aging results for *F. hygrometrica*.

Regarding the epiphytic species (*Ulota crispula*, *Lewinskya iberica*, and *L. acuminata*), their habitat characteristics and pioneer character further support the observed low longevities. Epiphytic substrates are frequently vertical and therefore unlikely to retain propagules effectively. Moreover, these environments are characterized by restricted and intermittent water availability and frequent desiccation–rehydration cycles [55,56,57], conditions that are not conducive to long-term spore preservation. It is therefore reasonable that these species do not form persistent spore banks in their substrate, which is consistent with the short longevities detected in our study.

The mechanisms responsible for the comparatively short longevity observed in the studied bryophyte spores remain poorly understood. However, the marked differences among species and maturation stages suggest that longevity is influenced by multiple factors. In the following sections, we discuss two non-mutually exclusive hypotheses that may account for these differences: the progressive acquisition of longevity during spore maturation and the potential influence of chlorophyll retention in mature spores.

### 3.2. Influence of Spore Maturity on Longevity

Examining P50 values in Table 1 and Table 2 reveals a clear upward trend in longevity between maturity levels in *Funaria hygrometrica* spores in both storage conditions, starting from the “green” or immature capsules and progressing to the “brown” or mature capsules. This trend was statistically significant only under dry storage conditions, which could indicate that the influence of maturity on spore longevity is stronger in dry than in accelerated aging conditions. On the other hand, the lack of statistical significance in the trend observed in the accelerated aging conditions could just indicate the need to select a more frequent sampling strategy in a system that aged very fast. We measured germination every 2 or 3 days and P50s ranged between 3.6 and 5.7 days. Nevertheless, this upward trend in longevity with spore maturity observed in both storage conditions resembles the increase in desiccation tolerance and longevity observed during seed maturation [6,7,14]. In seeds, the late maturation stage involves a reduction in the water content and an accumulation of protective molecules like Late Embryogenesis Abundant (LEA) proteins, raffinose family oligosaccharides (RFOs), and antioxidants, which in turn favor the vitrification of the cytoplasm and slow down natural degradation and loss of viability [6,7,14,58,59]. Indeed, during the late seed maturation stage, longevity is acquired gradually, increasing 30- to 50-fold to reach a maximum around the final dry and mature state [14]. The presence of a vitrified cytoplasm in dry mature bryophyte spores has been previously measured [19] but the underlying physiological, biochemical and structural changes occurring during spore maturation remain unknown in bryophyte spores and need further investigations.

Another explanation may also be possible in relation to a potential morphophysiological dormancy associated with incomplete spore development [60] that may be expressed during the storage experiments. Although not well characterized, some studies have suggested that dormancy may be present in some bryophyte spores, particularly from very hot and cold environments [60,61]. There are also recent studies that demonstrate some kind of smoke-induced dormancy breaking in *Sphagnum* spores [62,63,64]. Whether the low initial germination of some of our species (*L. iberica*, *U. crispula*) was due to some kind of dormancy is something we will have to consider in future experiments with complementary viability measures other than germination (e.g., efficiency of the photosynthetic apparatus of the spore chloroplast [27], tests of tetrazolium or fluorescein diacetate). However, we consider that it is quite improbable that some kind of secondary dormancy was activated only in immature *F. hygrometrica* spores in comparison to the more mature ones during both storage regimes, as all of them showed high (70–90%) germination percentages initially (Figure 3 and Figure 5).

Maturation drying and the acquisition of longevity in seeds also depends on the reduction in chlorophyll and the dismantlement of the chloroplasts [7,14]. We have observed that *F. hygrometrica* spores are released from mature capsules without the presence of chloroplasts ([19], personal observation). However, we do not have data to confirm that it is during *F. hygrometrica* spore maturation from green to brown capsules that chlorophyll contents are reduced and chloroplasts dismantled. On the other hand, the spores of the other species in our study appear to retain plastids when mature (personal observation using a fluorescent microscope as in [19]). Although we did not assess chlorophyll content, photosynthetic activity, or oxidative damage, this chlorophyllous condition could represent one possible explanation for their comparatively shorter lifespan. In seeds of vascular plants and fern spores, the persistence of active photosystems has been associated with increased oxidative stress and accelerated loss of viability [7,27,50,65]. Whether similar mechanisms operate in bryophyte spores remains to be investigated.

### 3.3. Implication for the Ex Situ Conservation of Bryophytes

Traditionally, bryophyte conservation has leaned on botanical garden collections or in vitro methods like cryopreservation [22]. Recent research emphasizes the suitability of storing bryophyte spores in spore banks at sub-zero temperatures following seed-like conditions (dried at 15–25% RH and stored at −20 °C) [19]. This is supported by our data, as *F. hygrometrica* spores slowed down the rate of viability loss regardless of the maturity stage when stored dry at 15% and 20 °C, as also observed in seeds and spores from other plant systems and many anhydrobiotes [66]. However, our study suggests a short longevity of the bryophyte spores, so potential fast decline in viability under standard storage conditions could be expected [19] as observed in other short-lived systems (e.g., chlorophyllous seeds and fern spores) [7,26,27]. Reducing storage temperature is known to increase seed and fern spore longevity by slowing down the molecular reactions responsible for aging, including oxidative damage and other deteriorative processes, while maintaining the cytoplasm in a stable glassy state in dry propagules [2,3,6,7,9,14,26]. Hence, storage of dry spores at lower sub-zero temperatures (−80 °C) or in cryogenic conditions in liquid nitrogen at −196 °C is expected to further suppress these aging processes. These conditions have proven to be more effective measures to improve long-term viability of short-lived seeds and spores [2,9], presenting, along with cryopreservation of gametophytic tissues [21,67], appealing alternatives to spore banking at conventional seed bank conditions [19].

## 4. Materials and Methods

### 4.1. Plant Materials

The present study was conducted on four different species from three different Bryophyte genera: *Funaria* (Funariaceae), *Ulota* and *Lewinskya* (Orthotrichaceae). Plant materials (gametophytes and sporophytes) were sourced from different localities in Spain, except for *U. crispula*, which was collected in Norway (Table 4). These species were selected due to their availability for spore collection at the time the experiment was planned, the possibility to easily select different maturity stages based on capsule color, in the case of *Funaria hygrometrica*, and the diversity of ecological features.

In all cases, different cushions or patches within the same population were collected and the sporophytes were kept attached to the gametophyte till the moment of the spore extraction. Drying was carried out on a laboratory bench, without the use of a drying oven.

*Funaria hygrometrica* is an ephemeral, pioneer, mostly terricolous species that grows in areas where there is or has been some kind of disturbance and it is a common weed in greenhouses. It is a cosmopolitan plant present on every continent except Antarctica thanks to its tolerance to a wide range of environmental conditions [68]. Spores are small and unicellular, 15–20 µm in diameter, spheroidal or ovulate, thin-walled and irregularly papillose. Their dispersal is xerochastic [68].

*Ulota crispula* is an epiphyte that grows on the trunks and branches of a wide variety of trees and shrubs. It shows a preference for temperate zones with oceanic to hyper-oceanic climates where humidity is higher [69], being widespread in eastern North America (southern Canada and the USA, except the southernmost areas), most of Europe (rare in the northernmost and Mediterranean areas) and southwestern Asia (Pontic Mountains, Caucasus) [69]. Spores are medium-sized, 20–29 µm in diameter, moderately thick-walled covered by thick papillae, with xerochastic dispersal [69].

*Lewinskya iberica* is an epiphyte that grows on the trunks, bases and branches of various trees and shrubs around the altitude of 580–1500 m in Mediterranean areas with some oceanic influence that softens the aridity. *L. iberica* is a species which normally requires a humid to sub-humid climate; however, it is adapted to grow in more xeric climates characterized by prolonged, intense summer droughts [70]. It grows together with other xerophilous and pioneer bryophytes, most commonly with other species within its family [71]. Spores are small and unicellular, (15–)19–22(–25) µm in diameter, sub-ellipsoidal in shape, generally slightly flattened, thin and densely papillose, irregularly ornamented by perineal elements leaving bare spots. It has hygrochastic dispersal [65,71].

*Lewinskya acuminata* is a Mediterranean epiphytic species well adapted to the seasonal aridity [72], frequently found on the trunks and branches of a variety of scrubland and forest communities. Although it is more frequent in more relatively humid climates of mountain areas, its elevational range varies from (0–)150 to 1850(–2100) m above sea level. It is distributed alongside the entire Mediterranean Region, even forming border communities within the Macaronesian Region. It has recently been discovered beyond the Mediterranean basin, in north-western and central Europe, but also disjunctly in California and Ethiopia through recent long-distance dispersals [17]. Spores are medium-sized, 20–28 µm in diameter, ellipsoidal in shape with a flattened pole, thin-walled and densely papillose, ornamented by verrucae and baculae. It presents hygrochastic dispersal [65,72].

### 4.2. Spore Extraction

Spores from the species in the Orthotrichaceae family were collected at the full maturity stage, when sporophyte capsules were completely developed and brown in color, but with the operculum still attached to the capsule. Spores of *Funaria hygrometrica* were collected at different maturity stages based on the color of the capsules: green (indicating spore immaturity), yellow (for intermediate spore maturity), and brown (full spore maturity).

The spores were obtained with the help of antistatic tweezers and a stereoscopic microscope by removing the operculated capsules from the plants and collecting them on an Eppendorf to avoid contamination. Then, capsule walls were crushed within the Eppendorf to release the spores. There was one Eppendorf for each species used in the study, except for *Funaria hygrometrica*, where we had three different Eppendorfs representing the different maturity stages the spores were collected at based on the colour of the capsules: green, yellow and brown.

### 4.3. Preparing the Culture Medium

All germination tests were conducted on Knop medium for bryophytes [73], composed of FeSO_4_·7H_2_O (12.5 mg/L), KH_2_PO_4_ (25 g/L), Ca(NO_3_)_2_ (100 g/L), KCl (25 g/L), MgSO_4_ (25 g/L) and solidified with 1.2% (p/V) (12 g/L) agar in Petri dishes of 5.5 cm diameter.

To prepare 1 L of culture medium, 10 mL was taken from the previously mentioned components, and distilled water was added to produce a volume of 1 L. Once the pH was adjusted to 5.8 with KOH (1 N), plant agar was added to the solution and then sterilized in an autoclave for 25 min (120 °C and 1 atm overpressure). The agar solution was poured into the Petri dishes prior to the solidification of the medium. The latter process was carried out in a laminar flow chamber to avoid contamination.

### 4.4. Accelerated Aging

Spores from the selected species were assessed using the controlled aging test according to the standard protocol used at The Millenium Seed Bank, Royal Botanic Gardens, Kew, UK [10], over a period of three months in 2023 in the Germplasm Laboratory in the University of Valencia Botanical Garden. The controlled aging test was conducted by placing these Eppendorf tubes with their lids open within airtight jars which contained a solution of LiCl (LiCl, 300 g/L) to generate a relative humidity (RH) of 60% and then they were placed in an oven set at 45 °C.

Spore samples were taken at the beginning of each experiment (t = 0) to sow three replicates in Petri dishes before storage under accelerated aging conditions, which were used as a reference for initial spore viability. The experiment continued until full loss of spore viability (last measurement for *F. hygrometrica* at 52 days and 11 days for *U. crispula*, *L. iberica* and *L. acuminata*).

Eppendorfs filled with 1 mL of distilled water were prepared in advance. Spores were collected by using the pipette tip to scrape off the spores and then depositing those that had adhered to the walls into the 1 mL of water to resuspend them and facilitate seeding. For each day of spore sampling, three replicate culture plates were prepared for each species/maturity stage, and so the 1 mL of water containing the spores was distributed equally (~330 µL) over the surface of the Petri dishes. Once the water had evaporated from the plates, thus leaving the spores on the medium, they were sealed with Parafilm and placed in a germination chamber at 20 °C with a 12 h photoperiod. This whole procedure is carried out within a flow chamber to avoid contamination of the medium.

Once sowed, the spores were observed with a stereoscopic microscope every day until germination was initially observed and then every few days subsequently (depending on germination rate). Each time the number of germinated spores and non-germinated spores were counted, with a total of 200 spores being counted for each dish. A spore was considered germinated if the length of the emerging protonema was equal to about half the diameter of the spore. Total germination for every day observed was pooled from the three replicates. Data was collected until the germination stabilized, i.e., the proportion of germinated spores did not change (less than 3% variation) for three consecutive days of observation, or when it was no longer possible to differentiate spores due to overgrown protonemata.

According to a modified classification of seed longevity [12], seed persistence can be categorized based on the P_50_ values obtained: P_50_ values of less than 2 days correspond to transient or ephemeral seeds; values of 2 to 10 days correspond to very short-lived seeds; P_50_ values of 10 to 100 indicate a medium longevity in seeds; values of 100 to 1000 are considered long-lived seeds; and values that exceed 1000 days are considered extremely long-lived. For our experiment concerning bryophyte spores, the same classification was applied.

### 4.5. “Dry Storage” Conditions

For the experiment involving *Funaria hygrometrica* stored at dry conditions (15% RH at 20 °C), the same procedure is followed but the spores are not previously stored in controlled aging conditions, since our objective is to observe the natural decline of viability in the plant. Initial samples were taken at t = 0 days before being placed in dry conditions, following the same procedure as specified earlier. Germination was monitored in the same way as in the accelerated aging experiment, with counts taken daily or every few days after each sowing, until germination stabilized. The experiment continued for 150 days.

Seeds or spores from other plant systems placed within dry storage conditions tend to prolong their viability. As such, larger intervals of time (irregularly, once every one or two months) were allowed between each observation in these conditions, as the same prolonged viability was expected to occur [19].

### 4.6. Statistical Analysis

A generalized linear model (GLM) with binomial error and probit link was fitted to the final germination, using storage period and species or maturity stage (depending on the experiment) as predictors. The Akaike information criterion (AIC) was used to identify the best-fitted GLM model by removing non-significant factors.

Probit analysis was carried out using R (version 4.3.0) in RStudio version 2023.03.0 + 386 [74] to estimate P_50_ (the time for viability to fall to 50%) through the fitting of the basic seed viability equation by Ellis and Robert:

v = Ki − (p/σ),(1)

where v is the viability in normal equivalent deviates (NED) at time p (days) in storage, Ki is a constant related to the estimated initial viability, and σ is the standard deviation of the normal distribution of seed deaths over time.

Specifically, the above equation was expressed as a generalized linear model (GLM) with a probit (cumulative normal distribution) link function as follows [75], with the R package viabilitymetrics (version 0.0.0.9100):

y = φ(v) = φ(Ki − (1/σ)),(2)

There were instances where the initial quality of the spores from some species used for the experiment were much lower than anticipated (≤85%); a new control viability (Cv) parameter was introduced to the model—as per the suggestion of Mead and Gray [48]—which considers the initial viability of the spore lot:

y = Cv × φ(v) = Cv × φ(Ki − (1/σ)),(3)

The values for the Cv parameter were obtained from the highest percentage of germinated spores at the beginning of the experiment (t = 0). The P_50_ (Ki × σ) calculated for the species that had an initial viability percentage inferior to 85% was calculated using Equation (3); for percentages ≥85%, Equation (2) was used.

Multiple ANOVAs were conducted to analyze the relationship between spore longevity and spore maturity in *Funaria hygrometrica* (both stored in dry conditions and in accelerated aging conditions) and between species from the accelerated aging procedure. To be able to perform an ANOVA test, the percentage data was arcsine transformed in Excel in order to fulfil the assumptions necessary to carry out the test (independence and normality). Through the Akaike information criterion (AIC) test we found the best-fitted ANOVA model by discarding factors that did not contribute significantly to the data. In cases where significance was observed, a post hoc Tukey’s Honestly Significant Difference (Tukey’s HSD) test was employed to pinpoint and determine which pairwise differences among group means are statistically significant, helping to identify where the significance lies in the multiple comparisons.

## 5. Conclusions

Overall, our results demonstrate that the spores of the bryophyte species studied in this work exhibit markedly short longevity compared to the spores and seeds of other plant systems. This limited longevity suggests a potential reduced capacity to form long-term soil spore banks, reinforcing the idea that many bryophytes rely more on effective dispersal than on persistent below-ground propagule reserves. Furthermore, under dry storage conditions, spore maturity significantly increased longevity, with fully mature spores surviving significantly longer than immature ones. In contrast, this effect was not detected under accelerated aging conditions, indicating that the influence of spore maturity on longevity perhaps depends on the storage regime. The possible contribution of chloroplast persistence to reduced longevity also highlights important physiological mechanisms that warrant further investigation. From an applied perspective, these findings carry significant implications for ex situ conservation. Given the rapid decline in viability under standard storage conditions, alternative strategies such as storage at lower sub-zero temperatures or cryopreservation may be required to ensure the long-term conservation of bryophyte genetic resources.

## Figures and Tables

**Table 1:** Initial viability constant (Ki), standard deviation (σ) and 50% half-life (P_50_) calculated from the viability equations of the four species and maturity stages (capsule colors in *F. hygrometrica*) stored at accelerated aging conditions (60% RH and 45 °C).

Species	Ki	σ	P_50_ (Ki × σ) Days
*U. crispula* *	0.88	0.50	0.44
*L. iberica* *	0.68	1.30	0.88
*L. acuminata*	0.57	2.65	1.51
*F. hygrometrica* (brown)	0.92	6.20	5.70
*F. hygrometrica* (yellow)	1.12	3.56	3.99
*F. hygrometrica* (green) *	0.56	6.44	3.61

* Spore aging data was adjusted to the modified Ellis and Roberts equation [48], considering the initial viability before starting the experiment.

**Table 2:** Initial viability constant (Ki), standard deviation (σ) and 50% half-life (P_50_) calculated from the viability equations of *Funaria hygrometrica* spores at different stages of maturity. Spores were stored under dry conditions (15% RH and 45 °C).

Capsule Color (Maturity)	Ki	σ	P_50_ (Ki × σ) Days
Green (immature) *	0.62	93.8	58.2
Yellow (almost mature) *	0.72	107.1	80.3
Brown (fully mature)	0.95	158.5	150.6

* Spore ageing data was adjusted to the modified Ellis and Roberts equation [48], considering the initial viability before starting the experiment.

**Table 3:** Vascular plant species and the P_50_ values of their seeds or spores extracted from the literature. The letter “^a^” denotes seeded species subjected to accelerated aging conditions (45 °C and 60% RH), which were extracted from [11]; the letter “^b^” denotes fern species subjected to dry storage conditions (20 °C and 15% RH) which were extracted from [26,49].

Species	P_50_ (Days)
*Calothamnus graniticus* Hawkeswood ^a,^*	256.3
*Silene gallica* L. ^a^	114.7
*Ficus exasperata* Vahl ^a^	76.6
*Ficus salicifolia* Vahl ^a^	33.8
*Caiophora lateritia* Klotzsch ^a^	4.3
*Streptocarpus cyaneus* S.Moore ^a^	0.9
*Pteris vittata* L. (non-chlorophyllous spores) ^b^	4781
*Christella dentata* (Forssk.) Brownsey & Jermy (non-chlorophyllous spores) ^b,^*	310
*Matteuccia struthiopteris* (L.) Tod. (chlorophyllous spores) ^b,^*	119
*Todea barbara* (L.) T.Moore (chlorophyllous spores) ^b^	15

* The species names are those that appear in the original literature from where data was collected; the current accepted names are *Melaleuca granitica* (Hawkeswood) Craven & R.D.Edwards, *Thelypteris dentata* (Forssk.) E.P.St.John, and *Onoclea struthiopteris* (L.) Roth for *C. graniticus*, *C. dentata* and *M. struthiopteris*, respectively.

**Table 4:** Summary of information regarding the specimens used in the study.

Species	Collection Date	Locality	Altitude	Capsules	Habitat
*Funaria hygrometrica*	21 December 2022	CIEF ^1^, Quart de Poblet, Valencia, Spain. 39°28′53.04″ N 0°26′21.839″ O	78 m	>50	In glasshouse, on soil in pots containing forest species seedlings.
*Ulota crispula*	9 August 2022	Nordnesparken public park, Bergen, Norway. 60°24′01.47″ N 5°18′05.18″ E	10 m	50	On trunks of *Fagus sylvatica* L. in a garden by the sea.
*Lewinskya iberica*	2 May 2021	Piornal, Cáceres, Spain. 40°06′55″ N 5°50′38″ O	1190 m	22	Collected from *Ligustrum lucidum* Ait. trunks growing beside a road.
*Lewinskya acuminata*	12 October 2022	Sierra de El Toro, Castellón, Spain. 39°58′51.668″ N 0°45′3.654″ O	1200 m	37	Trunks of *Pinus nigra* J.F.Arnold growing by the road.

^1^ CIEF: Centro para la Investigación y la Experimentación Forestal (Center for Forestry Research and Experimentation), Generalitat Valenciana.

## Data Availability

The original contributions presented in this study are included in the article. Further inquiries can be directed to the corresponding author.

## Abbreviations

The following abbreviations are used in this manuscript:

AA	Accelerated aging
RH	Relative humidity
P_50_	Half-life
